# Crucial Involvement of Tumor-Associated Neutrophils in the Regulation of Chronic Colitis-Associated Carcinogenesis in Mice

**DOI:** 10.1371/journal.pone.0051848

**Published:** 2012-12-18

**Authors:** Kun Shang, Yu-Pan Bai, Chen Wang, Zhen Wang, Hong-Yu Gu, Xiang Du, Xiao-Yan Zhou, Chun-Lei Zheng, Ya-Yun Chi, Naofumi Mukaida, Ying-Yi Li

**Affiliations:** 1 Cancer Research Laboratory, Fudan University Shanghai Cancer Center, Department of Oncology, Shanghai Medical College, Fudan University, Shanghai, China; 2 Division of Molecular Bioregulation, Cancer Microenvironment Research Program, Cancer Research Institute, Kanazawa University, Kanazawa, Japan; French National Centre for Scientific Research, France

## Abstract

Ulcerative colitis (UC) is a major form of chronic inflammation that can frequently progress to colon cancer. Several studies have demonstrated massive infiltration of neutrophils and macrophages into the lamina propria and submucosa in the progression of UC-associated colon carcinogenesis. Macrophages contribute to the development of colitis-associated colon cancer (CAC). However, the role of neutrophils is not well understood. To better understand the involvement of tumor-associated neutrophils (TANs) in the regulation of CAC, we used a mouse CAC model produced by administering azoxymethane (AOM), followed by repeated dextran sulfate sodium (DSS) ingestion. This causes severe colonic inflammation and subsequent development of multiple tumors in mice colon. We observed that colorectal mucosal inflammation became increasingly severe with AOM and DSS treatment. Macrophages infiltrated the lamina propria and submucosa, together with a marked increase in neutrophil infiltration. The chemokine CXCL2 increased in the lamina propria and submucosal regions of the colons of the treated mice, together with the infiltration of neutrophils expressing CXCR2, a specific receptor for CXCL2. This process was followed by neoplastic transformation. After AOM and DSS treatment, the mice showed enhanced production of metalloproteinase (MMP)-9 and neutrophil elastase (NE), accompanied by excessive vessel generation and cell proliferation. Moreover, CXCL2 promoted neutrophil recruitment and induced neutrophils to express MMP-9 and NE *in vitro*. Furthermore, administration of neutrophil-neutralizing antibodies after the last DSS cycle markedly reduced the number and size of tumors and decreased the expression of CXCR2, CXCL2, MMP-9, and NE. These observations indicate a crucial role for TANs in the initiation and progression of CAC and suggest that the CXCL2–CXCR2 axis might be useful in reducing the risk of UC-associated colon cancer.

## Introduction

Colorectal cancer (CRC) is one of the most common malignant neoplasms and a leading cause of death [1]. Epidemiological data suggest that infection and chronic inflammation contribute to approximately one in four of all cancer cases [2]. Ulcerative colitis (UC) is the primary constituent of inflammatory bowel disease [3], [4], manifesting as DNA damage with microsatellite instability in the colon, together with epithelial dysplasia, and eventually progressing to colon cancer [5]. Thus, the risk of CRC is higher in UC patients than in the general population [6]. Moreover, the duration and severity of UC correlate with the risk of developing colitis-associated colon cancer (CAC) [7]–[9].

Chronic UC mouse models are produced by supplying drinking water containing dextran sulfate sodium (DSS), which is widely employed to recapitulate the histological changes in the colons of UC patients [10]. Moreover, repeated colitis in mice induced by nine cycles of DSS administration contributes to the development of multiple intestinal tumors [11]. Azoxymethane (AOM) can also induce colon carcinogenesis in mice by mediating O^6^-methyl-guanine formation [12]. Administration of AOM followed by repeated DSS ingestion can cause a high incidence (almost 100%) of colon cancer [13]–[15]. Activation of the NF-κB pathway contributes to this colon carcinogenesis by preventing colon epithelial cell apoptosis and promoting growth factor production by inflammatory cells [16]. Chronic inflammation induces immune cells to express pro-inflammatory molecules such as interleukin (IL)-1α, tumor necrosis factor (TNF)-α, and IL-6. NF-κB is activated by TNF-α, followed by CCL2-CCR2 axis-mediated macrophages infiltration. This ultimately progresses to colon cancer [17]. Simultaneously, there is a massive infiltration of neutrophils into the lamina propria and submucosa in the progression of UC-associated colon carcinogenesis [17]. However, the roles of neutrophils in this neoplastic transformation are poorly understood.

Neutrophils have a major role in defending human hosts from invading microorganisms. They are the most common leukocyte type in the blood, constantly preparing to respond to chemotactic stimuli and fight infection [18]. Several studies have recently shown that neutrophils can synthesize a large number of serine proteinases, such as neutrophil elastase (NE), cathepsin G, proteinase-3, metalloproteinase 8 (MMP-8), and MMP-9, which alter the tumor microenvironment and promote tumorigenesis [19]. Neutrophils also express cytokines and chemokines, such as TNF-α, IL-1β, and the CC and CXC families [20]. CXCR2 is located on neutrophils and interacts with CXCL1, 2, 3, and 5 [21]. Furthermore, neutrophil-derived CXCL2 can enhance angiogenesis as well as neutrophil accumulation, ultimately inducing carcinogenesis [22]. Breast cancer cells can stimulate neutrophils to produce oncostatin M, which interacts with tumor cells to lead to the expression of vascular endothelial growth factor (VEGF), thereby augmenting tumor-associated angiogenesis [23]. In renal cell carcinoma, intratumoral neutrophils have been shown to be an independent predictor of mortality [24]. Neutrophil infiltration within alveolar spaces in bronchoalveolar cell carcinoma has been shown to be associated with a poor clinical outcome [25]. In colon cancer [26], [27], small cell lung carcinoma [28], and melanoma [29], an elevated neutrophil-to-lymphocyte ratio predicts a significantly higher risk of death. These observations suggest that tumor-associated neutrophils (TANs) play an indispensable role in the initiation and progression of CAC.

To better understand the crucial involvement of TANs in CAC regulation, we used a mouse model that received combined AOM and repeated DSS treatment. This led to the development of multiple tumors in the mice colon. Our experiments revealed that combined AOM and DSS treatment induced increasingly severe colorectal mucosal inflammation and increased CXCL2 expression, together with the infiltration of CXCR2-expressing neutrophils, in the lamina propria and submucosal regions of the colon. Moreover, after AOM and DSS treatment, the mice showed enhanced production of MMP-9 and NE, accompanied by excessive vessel regeneration and cell proliferation. CXCL2 promoted neutrophil recruitment and induced neutrophils to express MMP-9 and NE *in vitro*. Furthermore, TAN depletion with neutrophil-neutralizing anti-Ly6G antibodies markedly reduced the number and size of tumors and reversed tumorigenesis, even when colon carcinoma was already present.

## Materials and Methods

### Ethics Statement

This study was carried out in strict accordance with the recommendations in the *Guide for the Care and Use of Laboratory Animals* of Fudan University. The protocol was approved by the Committee on the Ethics of Animal Experiments of Fudan University (Permit Number, SYXK (Hu) 2009-0082). All surgeries were performed under sodium pentobarbital anesthesia, and all efforts were made to minimize suffering.

### AOM and DSS-induced CAC Mouse Model

Pathogen-free 6- to 8-week-old female WT BALB/C mice were purchased from Shanghai Medical College of Fudan University and housed in standard animal cages under specific pathogen-free conditions in the animal facility at the college. All mice were maintained according to the Institutional Animal Care Guidelines and were fed a regular basal diet and tapwater *ad libitum*. The mice were acclimated for 1 week and then randomly divided into 2 groups. In the treated group, the mice were intraperitoneally injected with 12 mg/kg body weight of AOM (Sigma-Aldrich, Inc.) dissolved in physiological saline. Five days after AOM administration, 2% DSS (MP Biomedicals, Inc.) was administered through the drinking water for 7 consecutive days. Subsequently, untreated water was given for 14 days. These 21 days constituted 1 cycle, and a total of 3 cycles were used. The control group was treated with physiological saline instead of AOM and DSS. During the course of the experiment, the mice were weighed 2 times weekly. The mice were sacrificed at the indicated time intervals for macroscopic inspection, total RNA and protein extraction, and histological analysis. In some experiments, anti-Ly6G antibodies (eBioscience, Inc.) or isotype-matched immunoglobulin G (IgG) antibodies (R&D Systems China Co. Ltd.) were injected into the tail veins of mice, daily from days 56–62. The mice were sacrificed on day 67, followed by total RNA and protein extraction and histological analysis. Blood was collected simultaneously with colon tissue removal. Blood counts were determined using an automated blood counter XE-2100 (ADICON, Shanghai, China).

### Histopathological and Immunohistochemical Analyses of Mouse Colon Tissues

Resected mouse colon tissues were fixed in 10% formalin neutral buffer solution for paraffin embedding. Paraffin-embedded tissues were cut into 4-µm sections and stained with hematoxylin and eosin (H&E) solution. Immunohistochemical staining for CXCR2, CXCL2, F4/80, MMP-9, CD31, and proliferating cell nuclear antigen (PCNA) was performed using sections from 7 randomly selected samples. Endogenous peroxidase activity was blocked with 3% H_2_O_2_ for 15 min. Antigen retrieval was performed by boiling the sections for 10 min in citrate buffer (pH 6.0) and cooling at RT, followed by blocking with 10% NGS for 1 h. The sections were incubated with optimal dilutions of anti-CXCR2 (Abcam), anti-CXCL2 (R&D),anti-F4/80 (AbD Serotec), anti-MMP-9 (Abcam), anti-CD31 (Abcam), and anti-PCNA antibodies (Cell Signaling Technology) overnight at 4°C. CXCR2^+^, CXCL2^+^, F4/80^+^, MMP-9^+^, and PCNA^+^ cells and CD31^+^ areas were detected with horseradish peroxidase (HRP)-conjugated anti-rabbit/mouse secondary antibodies using an EnViSion Detection Kit (Gene Tech Co. Ltd., Shanghai, China). The immune complexes were visualized using a Peroxidase Substrate DAB Kit (Gene Tech Co. Ltd., Shanghai, China) according to the manufacturer’s instructions. Finally, the slides were counterstained with hematoxylin and dehydrated, and cover slips were placed. Positive cells were counted in 5 randomly selected visual fields per section of each sample at 400× magnification. The CD31^+^ areas were also calculated in 5 randomly selected visual fields per section of each sample at 400× magnification using Adobe Photoshop CS5 software.

### Immunofluorescence Analysis of Mouse Colon Tissues

Immunofluorescence analysis was performed using eFluor® 625NC (eBioscience, Inc.) primary antibodies directed against mouse Ly6G, followed by examination using a fluorescence microscope (IX51; Olympus). For double-color immunofluorescence analysis, the sections were incubated separately with anti-CXCR2, anti-MMP-9, or anti-NE antibodies (Santa Cruz Biotechnology) at 4°C overnight and then with anti-mouse Ly6G eFluor® 625NC antibody. Goat anti-rabbit IgG fluorescein conjugate antibody (Calbiochem, Merck Serono Co. Ltd. China) was used as the secondary antibody for detecting CXCR2 and MMP-9. Donkey anti-goat IgG conjugate antibody was used as the secondary antibody for detecting NE. Immunofluorescence was visualized using the fluorescence microscope.

### RNA Isolation and cDNA Synthesis

Total RNA was extracted from part of the colon tissue using TRIzol reagent (Invitrogen Life Technologies, Carlsbad, CA, USA) according to the manufacturer’s instructions. After treatment with RNase-free DNase I (Promega Biotech Co., Ltd., Beijing, China), the RNA preparations were further purified using TRIzol LS reagent (Invitrogen) according to the manufacturer’s instructions. For primary peritoneal neutrophils, total RNA was extracted using the TRIzol LS reagent according to the manufacturer’s instructions. Total RNA (2 µg) was reverse transcribed into cDNA using a PrimeScript RT Reagent Kit (Takara Biotechnology Co., Ltd., Dalian, China) according to the manufacturer’s instructions.

### Semi-quantitative RT-PCR

To evaluate the amount of transcribed cDNA, serially 2-fold diluted cDNA products were amplified for glyceraldehyde 3-phosphate dehydrogenase (GAPDH) using the specific primer set (Table 1) for 25 cycles of 94°C for 30 s, 58°C for 30 s, and 72°C for 1 min in 25-µl reaction mixture containing Taq polymerase (Takara). Equal amounts of cDNA products were then amplified for the indicated genes using specific primer sets (Table 1), with an optimal cycle number of 94°C for 30 s, 55°C for 1 min, and 72°C for 1 min. The resultant PCR products were fractionated on a 1.5% agarose gel and visualized by ethidium bromide staining under ultraviolet transillumination. The band intensities were measured using ImageJ analysis software, and the ratios to GAPDH were calculated.

**Table 1:** Primer sequences for semi-quantitative RT-PCR.

	Sense (5′-…-3′)	Anti-sense (5′-…-3′)
CXCR2	CACCGATGTATACCTGCTGA	ACGCAGTACGACCCTCAAAC
CXCL2	GAACAAAGGCAAGGCTAACTGA	AACATAACAACATCTGGGCAAT
CXCL5	CCTCCTTCTGGTTTTTCAGTTTAGC	CCTCCTTCTGGTTTTTCAGTTTAG
MMP-9	AGTTTGGTGTCGCGGAGCAC	TACATGAGCGCTTCCGGCAC
NE	GGCGTGGGTGACAGAACTC	CGGTCTTTGGGATGGGTAA
GAPDH	TGTGATGGTGGGAATGGGTCAG	TTTGATGTCACGCACGATTTCC

### Quantitative RT-PCR

Real-time PCR was performed using an Applied Biosystems HT7900 PCR system with 2× QuantiFast SYBR Green PCR Master Mix (QIAGEN Co., Ltd., Shanghai, China), primers (1 µM; Table 2), and <100 ng cDNA in a 25-µl reaction mixture. Each target and standard GAPDH cDNA was analyzed in duplicate in three independent real-time RT-PCR assays. Thermal cycling was initiated with an activation step for 30 s at 95°C, followed by 40 cycles of 95°C for 5 s and 60°C for 30 s. Immediately after amplification, melt curve protocols were performed to ensure that primer dimers and other non-specific products had been minimized. Relative expression of target genes was analyzed by the ΔΔCt method. The ratios of mRNA levels were expressed relative to those of mRNA levels in the untreated control group. Results are expressed as mean ± SEM.

**Table 2:** Primer sequences for quantitative RT-PCR.

	Sense (5′-…-3′)	Anti-sense (5′-…-3′)
CXCL1	ACTGCACCCAAACCGAAGTC	TGGGGACACCTTTTAGCATCTT
CXCL2	ATCCAGAGCTTGAGTGTGACG	GTTAGCCTTGCCTTTGTTCAG
CXCL3	CCTACCAAGGGTTGATTTTGAG	GACTTGCCGCTCTTCAGTATCT
CXCL5	TTGATCGCTAATTTGGAGGTG	GCATTCCGCTTAGCTTTCTTT
GAPDH	TGTGTCCGTCGTGGATCTGA	CCTGCTTCACCACCTTCTTGA

### Immunoblotting Analysis

Colon tissues were homogenized and sonicated in RIPA lysis buffer (Beyotime Biotechnology, Suzhou, China) supplemented with protease and phosphatase inhibitors (Roche). After centrifugation at 20,000 *g* for 15 min, 50 µg of the supernatants were separated on a 12% SDS-polyacrylamide gel and transferred onto an Immunobilon-P transfer membrane (0.45 µm; Millipore). After blocking with 5% skim milk, the membranes were incubated with anti-phospho-Akt (1∶1000) and anti-Akt (1∶1000) antibodies (Cell Signaling Technology). Anti-β-actin antibody (1∶10000; Sigma) was used as an internal control. Goat anti-rabbit IgG-HRP and goat anti-mouse IgG-HRP antibodies (Santa Cruz Biotechnology) were used as secondary antibodies. The blotted membranes were treated using the SuperSignal West Dura Extended Duration Substrate (Pierce Biotechnology Inc.), and signals were detected using a Las-4000 mini CCD camera (GE Healthcare).

### Isolation and Culturing of Mouse Peritoneal Neutrophils

Eight- to 10-week-old female BALB/C mice were intraperitoneally injected with 2 ml 1% hepatin (Sigma) dissolved in physiological saline. After 3 h, peritoneal exudate neutrophils were harvested by lavage of the peritoneal cavity with 20 ml phosphate-buffered saline (PBS), centrifuged, washed, and collected. The resultant cell population was judged to be composed of 95% neutrophils after staining with Wright’s stain (Solarbio Co. Ltd., Beijing, China). The cell suspensions were centrifuged, plated in 6-well plates with RPMI 1640 containing CXCL2 (R&D), and incubated at 37°C for 3 h or 6 h to obtain total RNA for semi-quantitative RT-PCR. RPMI 1640 without CXCL2 was used as the control.

### Neutrophil Migration Assay

Neutrophils from the abdominal cavity were washed and resuspended in 2 ml PBS at a density of 1 × 10^6^/ml. RPMI 1640 containing the indicated concentrations of CXCL2 or 1 × 10^5^ neutrophils were placed in the lower and upper chambers (5-µm pore size filter; Corning), respectively, and incubated at 37°C for 2 h in 5% CO_2_. After cells remaining on the upper surface of the filters were removed mechanically, the cells that had migrated to the lower surface and bottom chamber were fixed with methanol and stained with Wright’s stain. The cell numbers were counted in >10 randomly selected fields for each filter at 200× magnification. RPMI 1640 without CXCL2 served as the control.

### Statistical Analysis

Data were analyzed using one-way ANOVA followed by the Fisher protected least significant difference test or Mann–Whitney U test. *p<0.05 was considered statistically significant.

## Results

### Established CAC Mouse Model by AOM and DSS Treatment and Inflammatory Cell Infiltration

**Figure 1:**
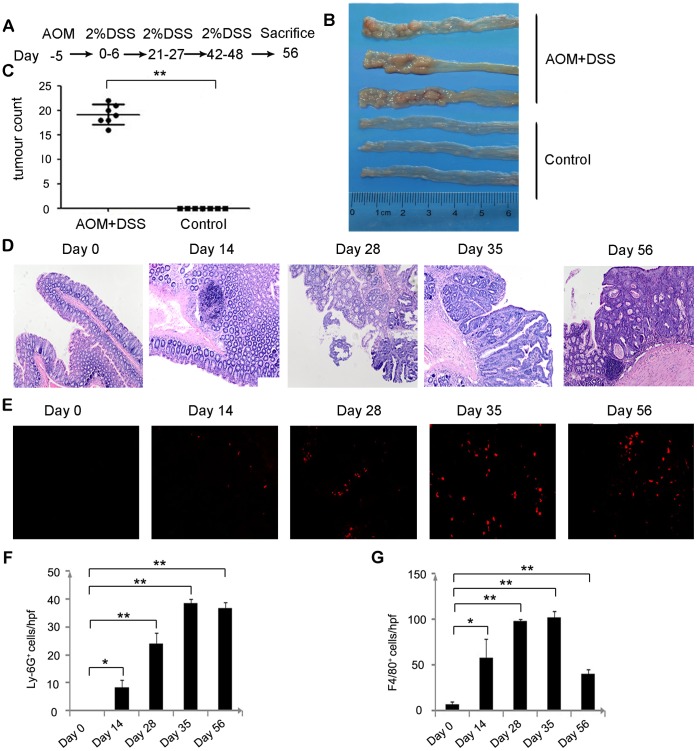
Tumor formation after AOM and DSS treatment and inflammatory cell infiltration. (**A**) Schematic overview of the colon carcinogenesis model. (**B**) Macroscopic changes in the colon. The colons were removed from the treated and control mice on day 56. Representative results of 7 mice per group are shown. (**C**) The numbers of macroscopic tumors in the colons removed on day 56 were determined. Each value represents mean ± SD (n = 7 mice/group). **p<0.01 versus control mice. (**D**) Colons were removed at the indicated times, fixed, and stained with H&E. Representative results of 7 treated mice are shown. Original magnification, 200×. (**E**–**G**) Colons removed from the AOM and DSS-treated mice at the indicated times were immunostained with anti-Ly6G or anti-F4/80 antibodies to determine the numbers of neutrophils (**E**, **F**) and macrophages (**G**), as described in the Materials and Methods. The cell numbers per field were counted in 5 randomly selected visual fields at 630× or 400× magnification. Representative results of 7 mice are shown. Each value represents mean ± SD (n = 7 mice/group). *p<0.05, **p<0.01 versus day 0 mice.

Previous reports showed that combining AOM with DSS could induce CRC [13]–[15]. In our study, a single intraperitoneal injection of AOM followed by 3 cycles of DSS ingestion resulted in the development of colon cancer (Figure 1A). The control group was administered physiological saline in place of AOM and DSS. During the course of the experiment, the treated mice exhibited profound body weight loss and bloody diarrhea, whereas the control mice exhibited no symptoms (data not shown). Macroscopic observations of the colons of all of the AOM and DSS-treated mice sacrificed on day 56 showed multiple tumors in the middle to distal colons (Figure 1B, C). Consistent with previous findings, H&E-stained colon tissues also showed tumor formation and atypical hyperplasia beginning on day 28 (Figure 1D). With time, an increasing number of inflammatory cells infiltrated the lamina propria and submucosa (Figure S1A). Immunofluorescence also revealed that mucosal infiltration of neutrophils increased, reaching the highest number on day 35 (Figure 1E, F). In addition, immunohistochemical analysis showed that the infiltrated macrophages became increasingly obvious, consistent with previous reports (Figure 1G and Figure S1B). These observations prompted us to investigate the role of neutrophils in UC-associated colorectal carcinogenesis.

### Enhanced CXCL2 and CXCR2 Expression during the Course of AOM and DSS-induced CAC

**Figure 2:**
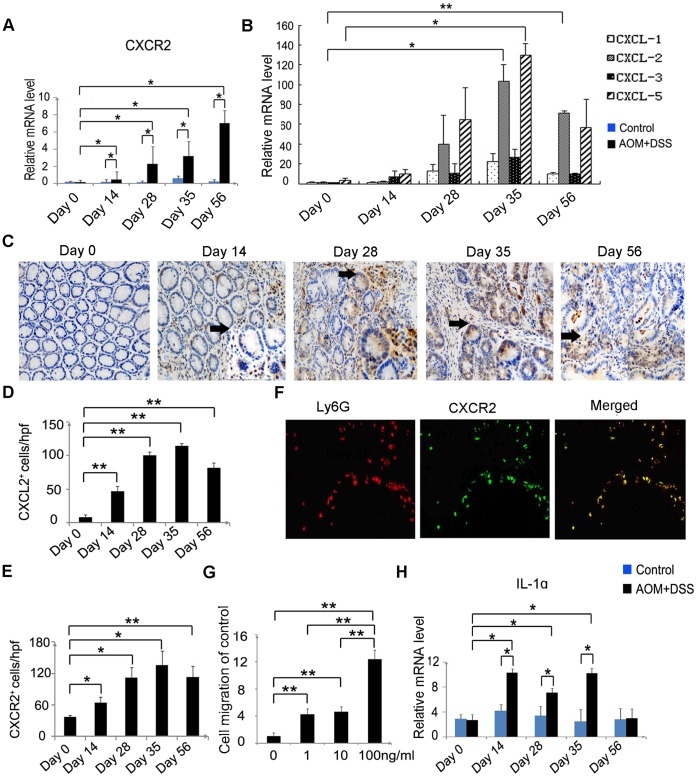
CXCR2 and CXCL2 expression in mouse colon tissues. *CXCR2* (**A**) and *CXCL1*, *CXCL2*, *CXCL3*, and *CXCL5* (**B**) mRNA expression in the colons of the two groups sacrificed at the indicated times was assessed by quantitative RT-PCR. Each value represents mean ± SD (n = 7 mice/group). *p<0.05, **p<0.01 versus control mice or day 0 mice. CXCL2^+^ (**C**, **D**) and CXCR2^+^ (**E**) cells were detected in the mouse colon tissues by immunohistochemical analysis. The colons were removed from the AOM and DSS-treated mice at the indicated times. Immunohistochemical analysis was performed using anti-CXCL2 and anti-CXCR2 antibodies, as described in the Materials and Methods. The cell numbers per field were counted in 5 randomly selected visual fields at 400× magnification. Representative results of 7 mice are shown in (**C**). Values in (**D**) and (**E**) represent mean ± SD for 7 mice in each group. *p<0.05, **p<0.01 versus day 0 mice. (**F**) Neutrophils expressing CXCR2 were detected by double-color immunofluorescence analyses using anti-Ly6G (red) and anti-CXCR2 (green) antibodies, as described in the Materials and Methods. The fluorescent images are digitally merged in the far right image. Representative results of 7 mice are shown. Original magnification, 630×. (**G**) The number of migratory Neutrophils in response to varying CXCL2 concentrations was counted in >10 randomly selected visual fields at 400× magnification. Values are mean ± SD of 3 independent experiments, expressed as fold increases over the control cells. **p<0.01 versus untreated cells. (**H**) Results of quantitative RT-PCR of *IL-1α* mRNA expression are shown. Each value represents mean ± SD (n = 7 mice/group). *p<0.05, **p<0.01 versus control mice or day 0 mice.

The recruitment of leukocytes to inflammatory sites is mediated by chemokines, and individual chemokines attract specific leukocyte populations through processes determined by ligand specificity and the expression patterns of the corresponding receptors [30]. Because CXCR2 is expressed by a proportion of neutrophils, we examined its expression in the colon tissues after AOM and DSS treatment. *CXCR2* mRNA was barely detectable in the colon tissues of the control mice. However, it was highly expressed in the colon tissues of the treated mice on day 28 and was further augmented until day 56 after AOM and DSS treatment (Figure 2A). We next evaluated mRNA expression of *CXCL1*, *CXCL2*, *CXCL3*, and *CXCL5*, the specific ligand for CXCR2. mRNA expression of *CXCL2,* and to lesser degree, *CXCL5*, but not *CXCL1* and *CXCL3*, had increased on day 28, peaked on day 35, and remained at a similar level until 56 days after AOM and DSS treatment (Figure 2B). CXCL2^+^ and CXCR2^+^ cells were increased (Figure 2C–E) and localized in the lamina propria and submucosa of the colon (Figure 2C and Figure S1C). Immunohistochemical analysis detected CXCL2 protein expression in infiltrating inflammatory cells and some tumor cells after AOM and DSS treatment (Figure 2C). Moreover, we observed that a substantial proportion of Ly6G^+^ neutrophils expressed CXCR2 (Figure 2F). Furthermore, we examined the effects of exogenous CXCL2 on neutrophils migration. We obtained pure mouse peritoneal neutrophils by hepatin stimulation (data not shown). The neutrophils migrated in response to CXCL2, and a positive correlation was observed between the number of migrated neutrophils and CXCL2 concentration (Figure 2G). These observations suggest that CXCL2 could induce neutrophils to infiltrate the inflammatory tissues by combining with CXCR2. In addition, we detected increased expression of pro-inflammatory *IL-1α* mRNA on day 14. The expression persisted until day 35 (Figure 2H). Therefore, it is possible that IL-1α overexpression led to increased CXCL2 expression, which eventually promoted neutrophil infiltration.

### Increased Neovascularization and Enhanced Cell Proliferation by Neutrophils Expressing MMP-9 and NE

**Figure 3:**
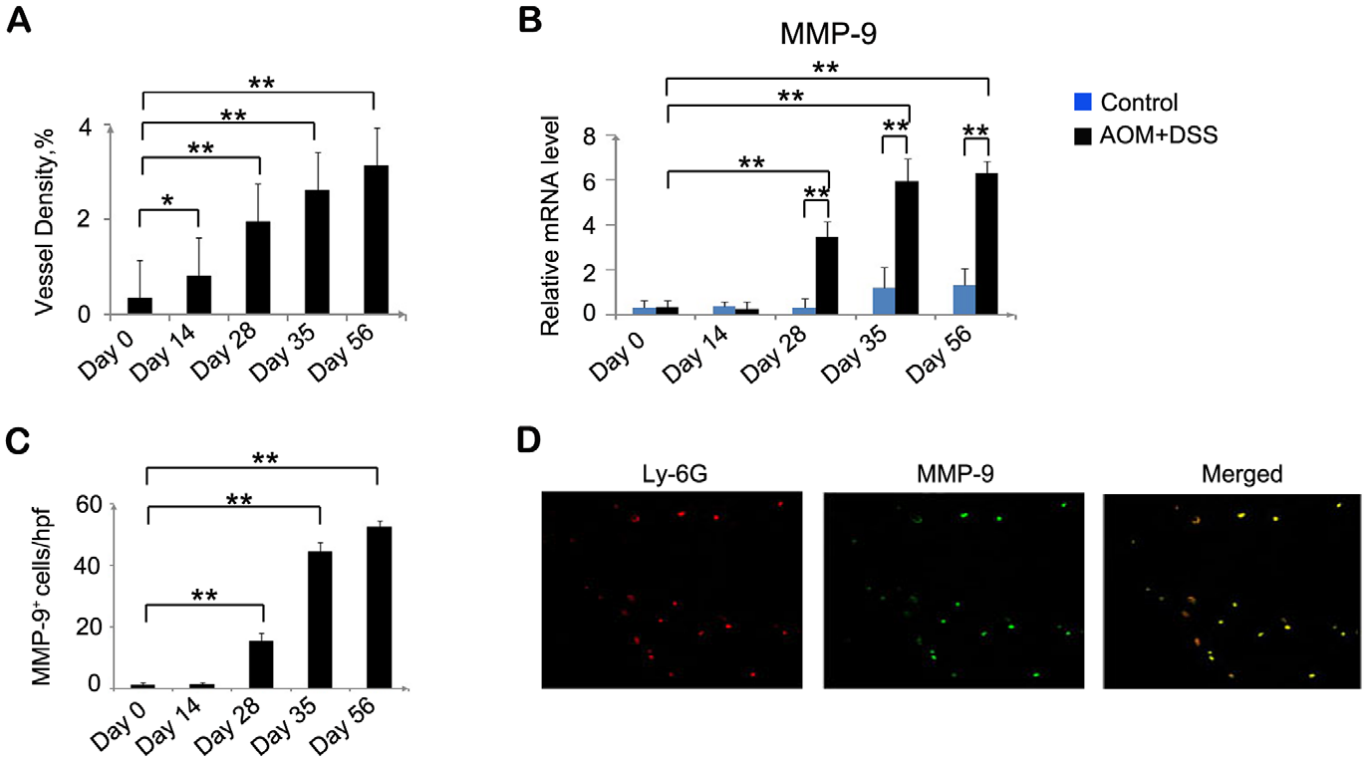
Vascular density and *MMP-9* expression. (**A**) Colon tissues were immunostained with anti-CD31 antibody, and vessel density was determined as described in the Materials and Methods. Each value represents mean ± SD (n = 7 mice/group). *p<0.05, **p<0.01 versus day 0 mice. (**B**) MMP-9 mRNA expression in the colons of the two groups sacrificed at the indicated times was assessed by quantitative RT-PCR. Each value represents mean ± SD (n = 7 mice/group). **p<0.01 versus control mice or day 0 mice. (**C**) Immunohistochemical detection of MMP-9^+^ cells in the mouse colon tissues. The colons were removed from the AOM and DSS-treated mice at the indicated times. Immunohistochemical analysis was performed using anti-MMP-9 antibodies as described in the Materials and Methods. The cell numbers per field were counted in 5 randomly selected visual fields at 400× magnification. Values represent mean ± SD for 7 mice in each group. **p<0.01 versus day 0 mice. (**D**) Neutrophils expressing MMP-9 were detected by double-color immunofluorescence analyses using anti-Ly6G (red) and anti-MMP-9 (green) antibodies, as described in the Materials and Methods. The fluorescence images are digitally merged in the far right image. Representative results of 7 mice are shown. Original magnification, 630×.

**Figure 4:**
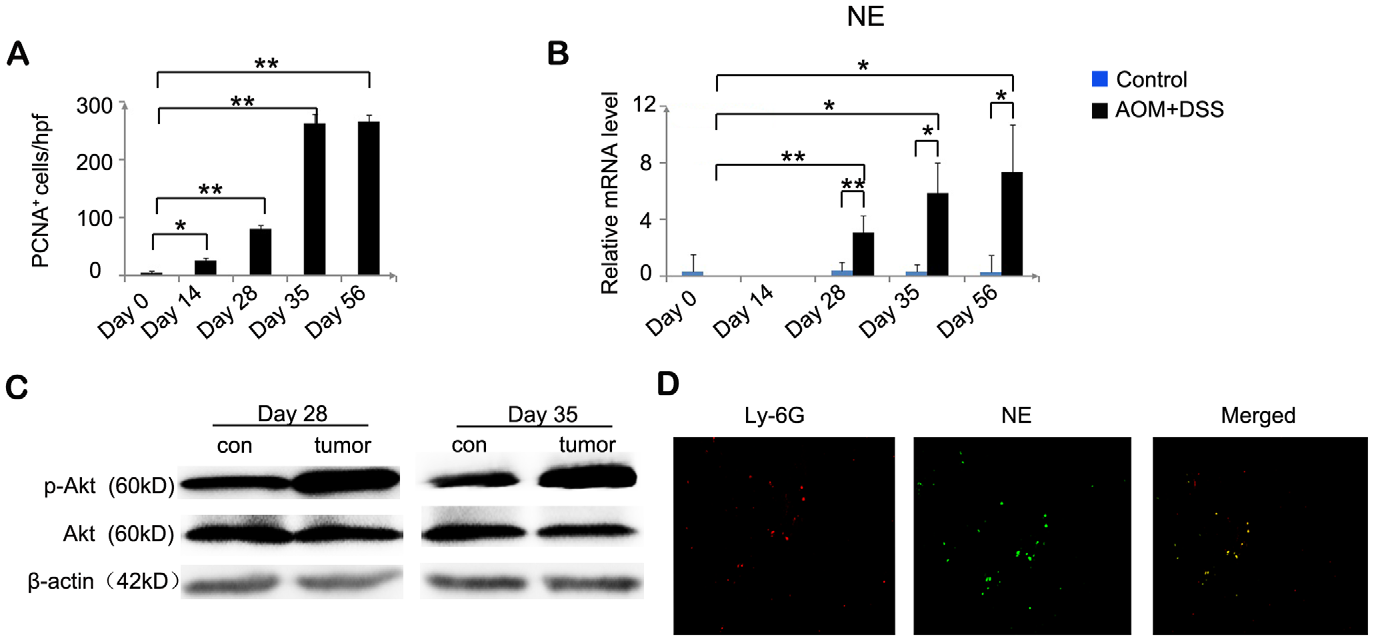
Increased expression of PCNA, NE, and pAKT^Ser473^. (**A**) PCNA^+^ cells were detected in the colon tissues. The colons were removed from the AOM and DSS-treated mice at the indicated times. Immunohistochemical analysis was performed using anti-PCNA antibodies as described in the Materials and Methods. The numbers of cells per field were counted in 5 randomly selected visual fields at 400× magnification. Values represent mean ± SD for 7 mice in each group. *p<0.05, **p<0.01 versus day 0 mice. (**B**) *NE* mRNA expression in the colons of the two groups sacrificed at the indicated times was assessed by quantitative RT-PCR. Each value represents mean ± SD (n = 7 mice/group). *p<0.05, **p<0.01 versus control mice or day 0 mice. (**C**) Immunoblotting analysis with anti-phospho-Akt (p-Akt) and anti-Akt (Akt) antibodies was performed using cell lysates of the colon tissues of the AOM and DSS-treated (tumor) and control (con) mice sacrificed on days 28 and 35, as described in the Materials and Methods. Representative results of 3 independent experiments are shown. β-actin served as a control for the mRNA levels. (**D**) Neutrophils expressing NE were detected by double-color immunofluorescence analyses using anti-Ly6G (red) and anti-NE (green) antibodies, as described in the Materials and Methods. The fluorescence images are digitally merged in the far right image. Representative results of 7 mice are shown. Original magnification, 630×.

**Figure 5:**
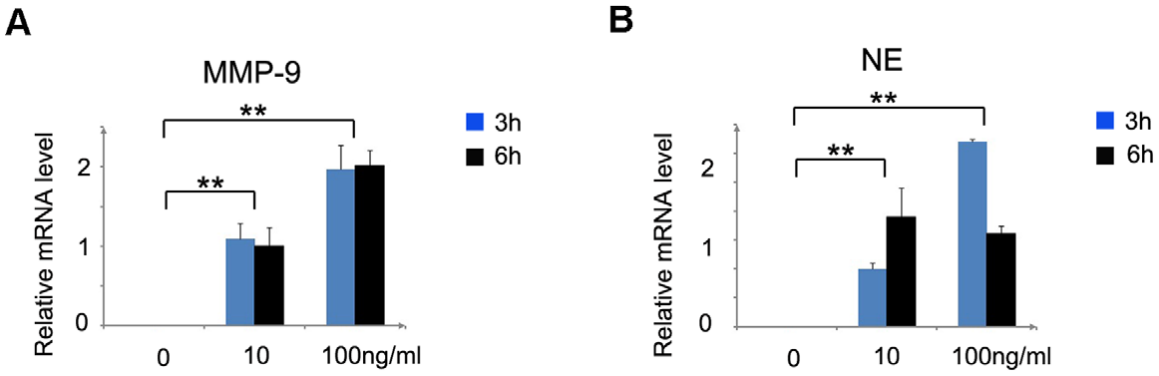
Migration of neutrophils and enhanced MMP-9 and NE expression by mouse peritoneal neutrophils in response to CXCL2. Total RNA was extracted from the mouse peritoneal neutrophils stimulated with different CXCL2 concentrations at the indicated times and subjected to RT-PCR analyses to determine MMP-9 (**A**) and NE (**B**) mRNA levels. Values represent mean ± SD of 5 individual experiments. **p<0.01 versus untreated cells.

Carcinogenesis is a complex, multistage process, which involves both angiogenesis and cell proliferation [31]. We evaluated angiogenesis at the sites of colon tumor foci by immunohistochemistry with anti-CD31. Intracolonic CD31^+^ vascular densities were markedly increased, reaching a maximal level on day 56 in the AOM and DSS-treated mice (Figure 3A and Figure S2A). Several lines of evidence suggest that neutrophils are potent initiators of angiogenesis because they have the ability to release MMP-9, an angiogenic factor [32], [33]. Therefore, we examined MMP-9 expression by quantitative RT-PCR and immunohistochemical analysis. *MMP-9* mRNA expression was remarkably enhanced, and the number of MMP-9^+^ cells also increased from day 28 to day 56 in the treated mice (Figure 3B, C, and Figure S2B). Moreover, double-color immunofluorescence analysis showed that Ly6G^+^ neutrophils could express MMP-9 (Figure 3D). We also examined PCNA expression. Although PCNA^+^ epithelial cells were barely detected in the control, PCNA expression was markedly increased in the nuclei of tumor cells after AOM and DSS treatment (Figure 4A and Figure S2C), suggesting that excessive proliferation of tumor cells ultimately led to the development of colon cancer. NE is a neutrophil-derived proteinase, which directly induces tumor cell proliferation by activating the Akt pathway [18]. Therefore, we next analyzed the expression level of NE and Akt phosphorylation. Quantitative RT-PCR for *NE* showed that *NE* mRNA expression was remarkably increased from day 28 to day 56 in the treated mice (Figure 4B). Moreover, Akt phosphorylation at Ser473 was enhanced on days 28 and 35 in the treated mice compared with the control mice, although no apparent differences were observed in Akt expression (Figure 4C). Furthermore, double-color immunofluorescence analysis showed that Ly6G^+^ neutrophils could express NE (Figure 4D). In addition, quantitative RT-PCR showed that CXCL2 could stimulate neutrophils to express MMP-9 and NE in vitro (Figure 5A, B). These observations suggest that neutrophils induce colon carcinogenesis through tumor-associated angiogenesis by MMP-9 and cell proliferation by NE.

### Reduced Tumor Formation by anti-Ly6G Antibody Administration

**Figure 6:**
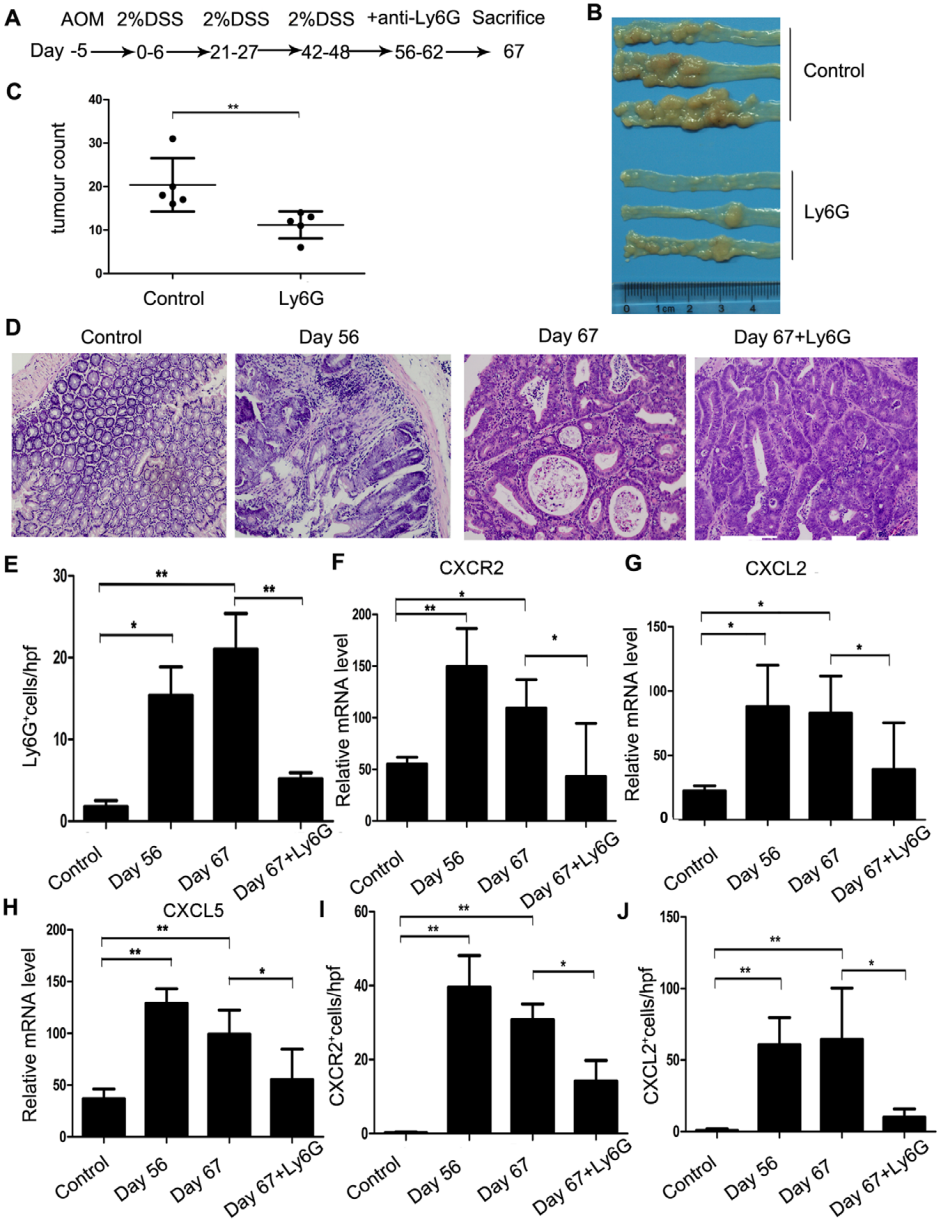
Effects of anti-Ly6G antibodies on colon carcinogenesis. (**A**) Schematic overview of anti-Ly6G antibody administration. Colons were removed on day 67 after the mice were administered anti-Ly6G antibodies or a vehicle control from day 56 to day 62. (**B**) Colons were removed on day 67 from the mice treated with anti-Ly6G antibodies or a vehicle control, and tumors were evaluated macroscopically. Representative results of 5 mice are shown. (**C**) The numbers of macroscopic tumors in the colons removed on day 67 were determined. Each value represents mean ± SD (n = 5 mice/group). **p<0.01 versus mice treated with the vehicle control. (**D**) Colons were processed for H&E staining, and representative results of 5 mice are shown. Original magnification, 200×. (**E**) Ly6G^+^ neutrophils were counted as described in the Materials and Methods. Values represent mean ± SD of the number of Ly6G^+^ neutrophils per field from 5 mice. *p<0.05, **p<0.01 versus control mice or mice treated with the vehicle control. (**F**–**H**) Quantitative RT-PCR analyses for *CXCR2* (**F**), *CXCL2*(**G**), and *CXCL5* (**H**) were performed on total RNA extracted from colons at the indicated times as described in the Materials and Methods. *CXCR2*, *CXCL2*, and *CXCL5* mRNA levels were normalized with *GAPDH* mRNA levels. *p<0.05, **p<0.01 versus mice treated with the vehicle control. (**I** and **J**) CXCR2^+^ (**I**) and CXCL2^+^ (**J**) cells were detected by immunohistochemical analyses of mouse colon tissues. The colons were removed on day 67 from the mice treated with anti-Ly6G antibodies or a vehicle control. Analysis was performed using anti-CXCR2 or anti-CXCL2 antibodies as described in the Materials and Methods. The numbers of cells per field were counted in 5 randomly selected visual fields at 400× magnification. Values are mean ± SD for 5 mice in each group. *p<0.05, **p<0.01 versus control mice or mice treated with the vehicle control.

**Figure 7:**
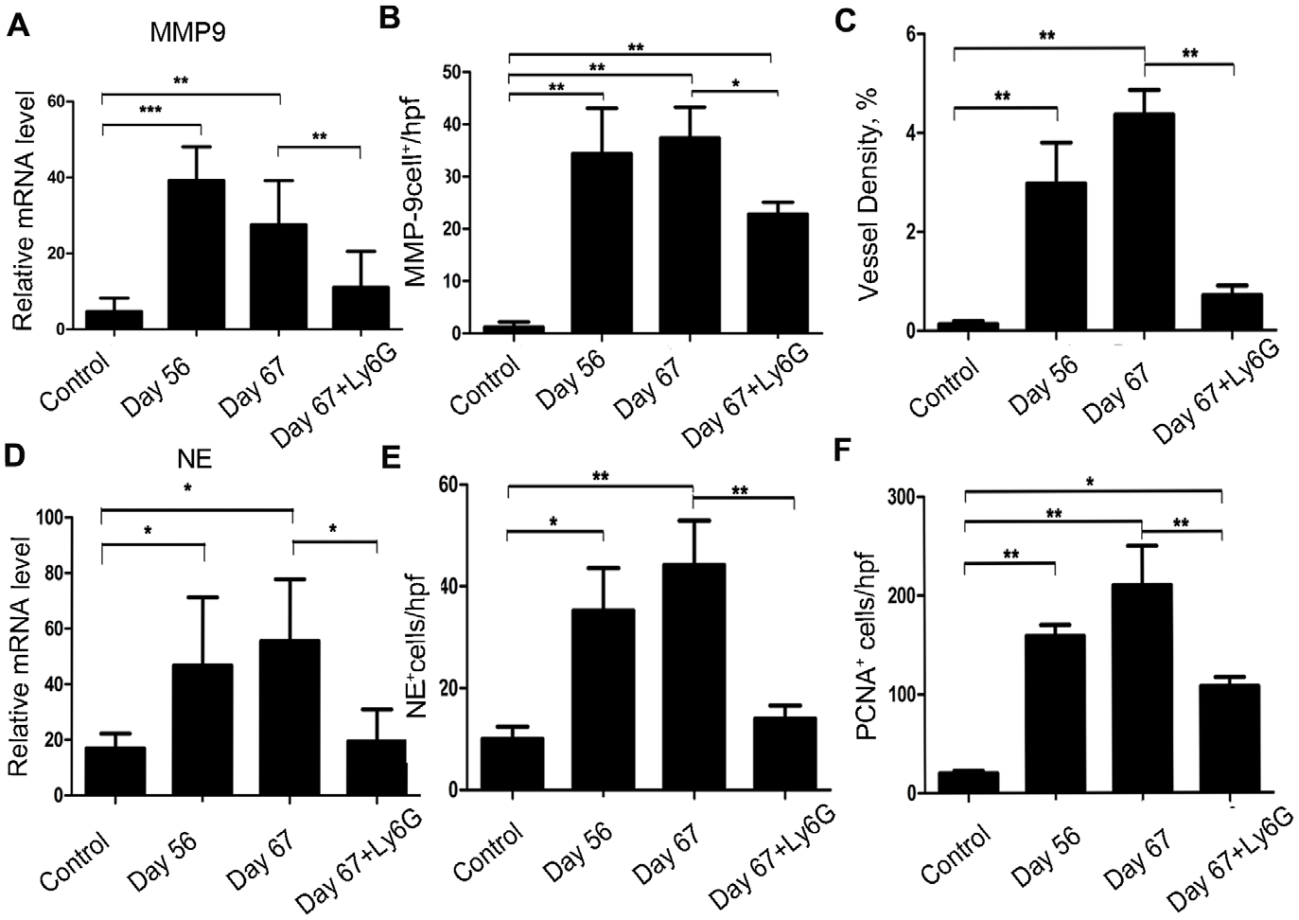
Effects of anti-Ly6G antibodies on MMP-9, NE, and PCNA expression and neovascularization. (**A**) *MMP-9* mRNA expression in the colon tissues from the mice treated with anti-Ly6G antibodies or the vehicle control was assessed by quantitative RT-PCR. Values are mean ± SD for 5 mice in each group. (**B**) Immunohistochical analysis was performed using anti-MMP-9 antibodies as described in the Materials and Methods. The cell numbers per field were counted in 5 randomly selected visual fields at 400× magnification. Values are mean ± SD for 5 mice in each group. *p<0.05, **p<0.01 versus control mice or mice treated with the vehicle control. (**C**) Colon tissues were immunostained with anti-CD31 antibody, and vessel density was determined as described in the Materials and Methods. Values are mean ± SD (n = 5 mice/group). **p<0.01 versus untreated control mice or mice treated with the vehicle control. (**D**) *NE* mRNA expression in the colons of mice treated with anti-Ly6G antibodies or the vehicle control was assessed by quantitative RT-PCR. Values are mean ± SD for 5 mice in each group. *p<0.05, **p<0.01 versus untreated control mice or mice treated with the vehicle control. (**E**) NE immunohistochemical analysis was performed using anti-NE antibodies as described in the Materials and Methods. The cell numbers per field were counted in 5 randomly selected visual fields at 400× magnification. Values are mean ± SD for 5 mice in each group. *p<0.05, **p<0.01 versus untreated control mice or mice treated with the vehicle control. (**F**) PCNA^+^ cells in the mouse colon tissues were detected by immunohistochemical analysis using anti-PCNA antibodies, as described in the Materials and Methods. The cell numbers per field were counted in 5 randomly selected visual fields at 400× magnification. Values represent mean ± SD (n = 5 mice/group). *p<0.05, **p<0.01 versus untreated control mice or mice treated with the vehicle control.

To further delineate the role of TAN interactions in the progression phase of this colon carcinogenesis model, we administered anti-Ly6G antibodies or a vehicle control to mice from day 56 to day 62 after AOM and DSS treatment (Figure 6A). There is no significant difference about the numbers of peripheral blood neutrophils between Anti-Ly6G antibody treatment and control IgG-treated mice (data not shown). Moreover, anti-Ly6G antibody treatment remarkably reduced the numbers of macroscopic tumors compared with control IgG treatment group, even when administered over this short and delayed time period (Figure 6B, C). Histopathological analysis showed that anti-Ly6G antibody treatment attenuated adenocarcinomatous changes (Figure 6D). Concomitantly, anti-Ly6G antibody treatment reduced intracolonic infiltration of inflammatory cells, especially neutrophils (Figure 6E and Figure S3A), together with decreases in *CXCR2*, *CXCL2*, and to lesser degree, *CXCL5* mRNA expression and the numbers of CXCR-2^+^ and CXCL2^+^ cells. (Figure 6F–J and Figure S3B, C). Moreover, anti-Ly6G antibodies reduced *MMP-9* mRNA expression (Figure 7A) and the numbers of MMP-9-expressing cells (Figure 7B and Figure `A). Furthermore, anti-Ly6G antibody treatment significantly decreased intratumoral CD31^+^ neovascularization (Figure 7C and Figure S4B). In addition, anti-Ly6G antibody treatment decreased *NE* mRNA expression in neutrophils and the numbers of NE^+^ cells (Figure 7D, E and Figure S4C). Finally, PCNA^+^ epithelial cells were significantly reduced by anti-Ly6G antibody treatment (Figure 7F and Figure S4D). These observations suggest that TAN depletion could reverse tumorigenesis, even when colon carcinoma was already present, probably by reducing neutrophil infiltration, which are a source of MMP-9 and NE that are involved in both tumor neovascularization and tumor cell proliferation.

## Discussion

This study was conducted to clarify the crucial role of TANs in the initiation and progression of CAC. Several studies have demonstrated that massive numbers of neutrophils and macrophages infiltrate the lamina propria and submucosa in DSS-mediated acute colitis and in the progression of UC-associated colon carcinogenesis in mice [34], [35]. Moreover, macrophages contribute to CAC development. However, the role of neutrophils has not been well understood, raising the possibility that neutrophils may also play a major role in chronic colitis-associated colon carcinogenesis. Hence, we examined the progression of chronic colitis-associated colon carcinogenesis using a CAC mouse model and anti-Ly6G antibodies and demonstrated the critical role of neutrophils in colon carcinogenesis.

This study utilized a well-accepted animal model of CAC [36], [37]. Oral administration of DSS solution to rodents produces a reliable UC mode that is widely employed to recapitulate the histological changes in the colons of UC patients [10]. Moreover, AOM administration followed by repeated DSS ingestion results in a high incidence (almost 100%) of colon cancer [13]–[15]. Therefore, AOM treatment followed by three cycles of DSS was used in our study. This resulted in the successful establishment of CAC. We showed that inflammation of the colorectal mucosa became increasingly severe in the treated mice. The structure of the lamina propria and submucosa was destroyed, and gland morphology was lost, resulting in neoplastic transformation.

TANs have been proposed as key mediators of tumor progression and angiogenesis [18], [38]. Accumulating evidence suggests that neutrophil infiltration of tumors may be associated with poor clinical outcomes for metastatic and localized renal cell carcinoma [24], [39], human hepatocellular carcinoma [40], head and neck squamous cell carcinomas [41], and bronchoalveolar carcinomas [25], [42]. Moreover, neutrophil infiltration correlates with tumor grade in human gliomas [43] and with more aggressive types of pancreatic tumors [44]. In our experiments, we observed markedly increasing numbers of neutrophils infiltrating into the lamina propria and submucosa, together with macrophage infiltration, in the treated mice. Moreover, anti-Ly6G antibody treatment attenuated intracolonic neutrophil infiltration and reduced the numbers of colon carcinomas. These observations suggest that neutrophil infiltration may play a key role in CAC development under our conditions.

Neutrophils defend humans from invading microorganisms through the immune response. In recent years, however, it has become obvious that neutrophils have other roles. Indeed, neutrophils can be induced to express an array of genes, including those encoding complement components, Fc receptors, and various chemokines and cytokines [20]. It is well known that leukocytes infiltrate injured tissues. This infiltration is induced by inflammation and chemokines play a key role in it [45]. Because of ligand specificity, different leukocytes accumulate in the inflammatory tissues, where they play important roles [45]. In mice, neutrophils express CXCR2, a specific receptor for GRO-α,β,γ/CXCL1,2,3 and epithelial cell-derived neutrophil-activating peptide-78 (ENA-78)/CXCL5 [46], [47]. The CXCR2 macromolecular signaling complex can regulate neutrophil mobilization, chemotaxis, and transepithelial migration [48]. Moreover, CXCR2 ligands chemoattract pro-tumoral neutrophils in esophageal and gastric cancers [46]. Furthermore, (ENA-78/CXCL5 binds CXCR2 and regulates the IL-17/granulocyte colony-stimulating factor axis and neutrophil homeostasis in mice [46]. In our experiments, *CXCL2* mRNA expression, and to a lesser degree, *CXCL5* expression increased in the treated mice, but *CXCL1* and *CXCL3* expression did not increase. As a consequence, we speculate that neutrophil infiltration may be mediated by the CXCL2–CXCR2 axis in the CAC model. Our data showing that neutrophils expressed CXCR2 in our mouse model are consistent with those of previous studies [49], [50]. Moreover, CXCL2 and CXCR2 expression were markedly increased in the AOM and DSS-treated mice. Furthermore, anti-Ly6G antibody treatment reduced the expression of CXCL2 and CXCR2. These observations were further confirmed by our evidence that CXCL2 induced neutrophil recruitment *in vitro*. CXCL2 was not only the first chemokine shown to be produced by neutrophils but was also shown to mediate neutrophil recruitment, release of granule enzymes, and expression of adhesion molecules by its interaction with neutrophils [51]. These findings suggest that CXCL2 was excessively expressed during inflammation and was chemotactic for neutrophils in combination with CXCR2. This alters the expression of oncogenes and cancer suppressor genes, thereby resulting in colon carcinogenesis.

MMP-9 is a widely expressed enzyme produced by many cell types including neutrophils [52]. MMP-9 has a role in promoting carcinogenesis, as confirmed in models of lung metastasis, pancreatic islet carcinoma, and skin carcinogenesis [32], [33]. Moreover, inflammatory cell-derived MMP-9 can drive tumor-associated angiogenesis by releasing VEGF [53]. Many proteinases have the ability to release VEGF. However, the role of MMP-9 is more remarkable in this regard [54]. As a result, we presumed that neutrophils promoted the development of multiple colon tumors by augmenting tumor-associated angiogenesis. We observed an increasing MMP-9 expression and neovascularization. Moreover, neutrophils expressed MMP-9 in our mouse model. These findings were further confirmed by the evidence that CXCL2 could induce neutrophils to express MMP-9 *in vitro*. In addition, anti-Ly6G antibody treatment reduced *MMP-9* mRNA expression and neovascularization. These observations suggest that neutrophil-derived MMP-9 drives tumor-associated angiogenesis and support the possibility that neutrophils promote CAC development.

NE is a key enzyme for tissue injury caused by activated neutrophils and possesses broad substrate specificity [55]. Moreover, NE is very neutrophil specific [56]. NE promotes lung carcinogenesis in the mouse model of lung adenocarcinoma by inducing cell proliferation and ultimately tumor growth [57]. NE enters tumor cell endosomes, degrading insulin receptor substrate-1 and releasing PI3Kp85. PI3Kp85 combines with platelet-derived growth factor receptor, increasing bioactive PI3K, which results in Akt phosphorylation and induction of cell proliferation [18]. In our mouse model, we showed that NE expression was significantly increased over time, together with an increase in phosphorylated Akt (pAkt) and PCNA. These data were further corroborated by the observation that CXCL2 could stimulate neutrophils to express NE *in vitro*. In addition, anti-Ly6G antibody treatment reduced *NE* mRNA expression and PCNA^+^ tumor cells. These observations suggest that neutrophil-derived NE activates PI3K/Akt signaling, enhancing tumor cell proliferation and the eventual progression to colon cancer.

Several studies have shown that colon mucosal epithelial cells are injured after stimulation and that microorganisms invade the mucosal and submucosal regions of the colon, leading to the accumulation of immune cells including neutrophils and macrophages. These immune cells release pro-inflammatory molecules, such as TNF-α, IL-6, and IL-1α, which activate signaling pathways and promote tumor growth. IKKβ/NF-κB plays an important role in CAC [16]. Knocking out IKKβ in mucosal epithelial cells could increase apoptosis, but it did not affect tumor size and inflammation. In immune cells with depleted IKKβ, however, tumor size and inflammation were reduced without affecting apoptosis [16]. NF-κB was activated by endogenously produced TNF-α in inflammatory cells, causing colonic inflammation and ultimately neoplastic transformation. We showed that the expression of pro-inflammatory IL-1α was increased, raising the possibility that NF-κB also might be activated by IL-1α, followed by increase in CXCL2 and infiltration of CXCR2-expressing neutrophils, with colon cancer developing from tumor-associated angiogenesis and cell proliferation.

In summary, our studies revealed for the first time the crucial involvement of TANs in promoting the initiation and progression of chronic colitis-associated colon carcinogenesis. Moreover, TAN depletion using anti-Ly6G antibodies reversed carcinoma progression, even after colon carcinoma was established. Considering the deleterious role of TANs in tumor progression, targeting polymorphonuclear neutrophils (PMNs) could be a new anti-cancer tool. However, PMNs are essential components of host defense against infectious agents. Therefore, targeting the CXCL2–CXCR2 axis might be effective for the treatment of colon cancer in individuals with UC.

## Supporting Information

Figure S1 Inflammatory cell infiltration after AOM and DSS treatment. (**A**) Colons were removed at the indicated times, fixed, and stained with hematoxylin and eosin. (**B**) Colons were removed at the indicated times and immunostained with anti-F4/80 antibodies to determine the numbers of macrophages. (**C**) Immunohistochemical analysis was performed using anti-CXCR2 antibodies as described in the Materials and Methods. Representative results of 7 mice are shown. Original magnification was 200× or 400×. Insets are 400× magnifications of areas indicated by arrows.
(TIF)

Figure S2 (**A**) Colon tissues were immunostained with anti-CD31 antibody. Representative results of 7 AOM and DSS-treated mice are shown. (**B**) Immunohistochemical analysis was performed using anti-MMP-9 antibodies as described in the Materials and Methods. Original magnification, 400×. (**C**) Representative results of immunohistochemical staining for PCNA in the colon tissues of 7 mice. Original magnification, 400×.
(TIF)

Figure S3 The effects of anti-Ly6G antibodies on neutrophil infiltration and CXCR2 and CXCL2 expression. Colon tissues were immunostained with anti-Ly6G (**A**), anti-CXCR2 (**B**), and anti-CXCL2 (**C**) antibodies as described in the Materials and Methods. Representative results of 5 mice are shown. (**B** and **C**) Original magnification, 400×.
(TIF)

Figure S4 The effects of anti-Ly6G antibodies on neovascularization and cell proliferation. Cells were immunohistochemically stained with anti-MMP-9 (**A**), anti-CD31 (**B**), anti-PCNA (**C**), and anti-NE antibodies (**D**). Representative results of 5 mice are shown. Original magnification, 400×.
(TIF)
