# Supplementary figures and images for: Crucial Involvement of Tumor-Associated Neutrophils in the Regulation of Chronic Colitis-Associated Carcinogenesis in Mice
Source: PLoS One. 2012 Dec 18;7(12):e51848. doi: 10.1371/journal.pone.0051848 (PMC3525572; doi:10.1371/journal.pone.0051848)

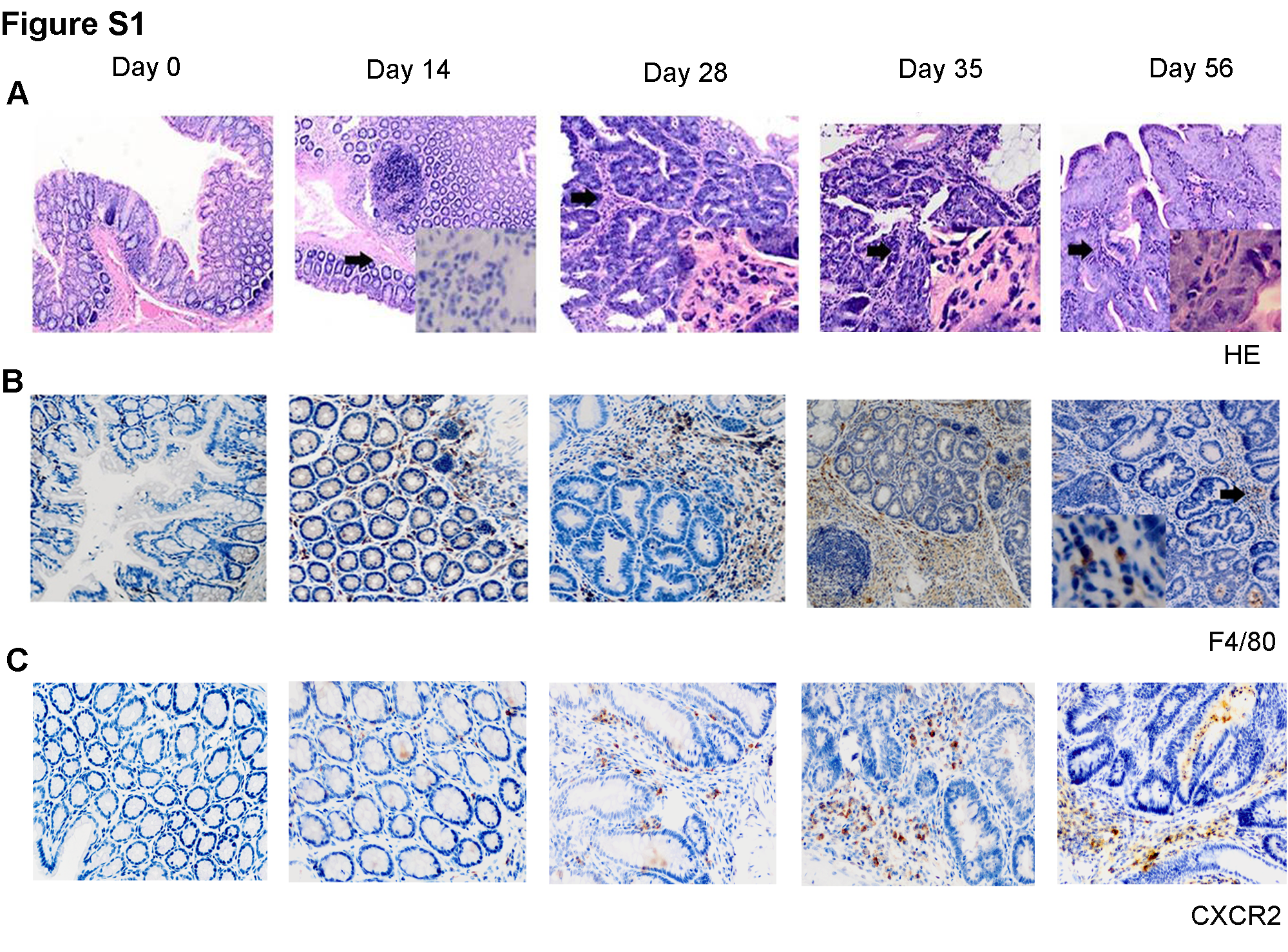

Supplement: Figure S1 — Inflammatory cell infiltration after AOM and DSS treatment. (A) Colons were removed at the indicated times, fixed, and stained with hematoxylin and eosin. (B) Colons were removed at the indicated times and immunostained with anti-F4/80 antibodies to determine the numbers of macrophages. (C) Immunohistochemical analysis was performed using anti-CXCR2 antibodies as described in the Materials and Methods. Representative results of 7 mice are shown. Original magnification was 200× or 400×. Insets are 400× magnifications of areas indicated by arrows. (TIF) [file pone.0051848.s001.tif]

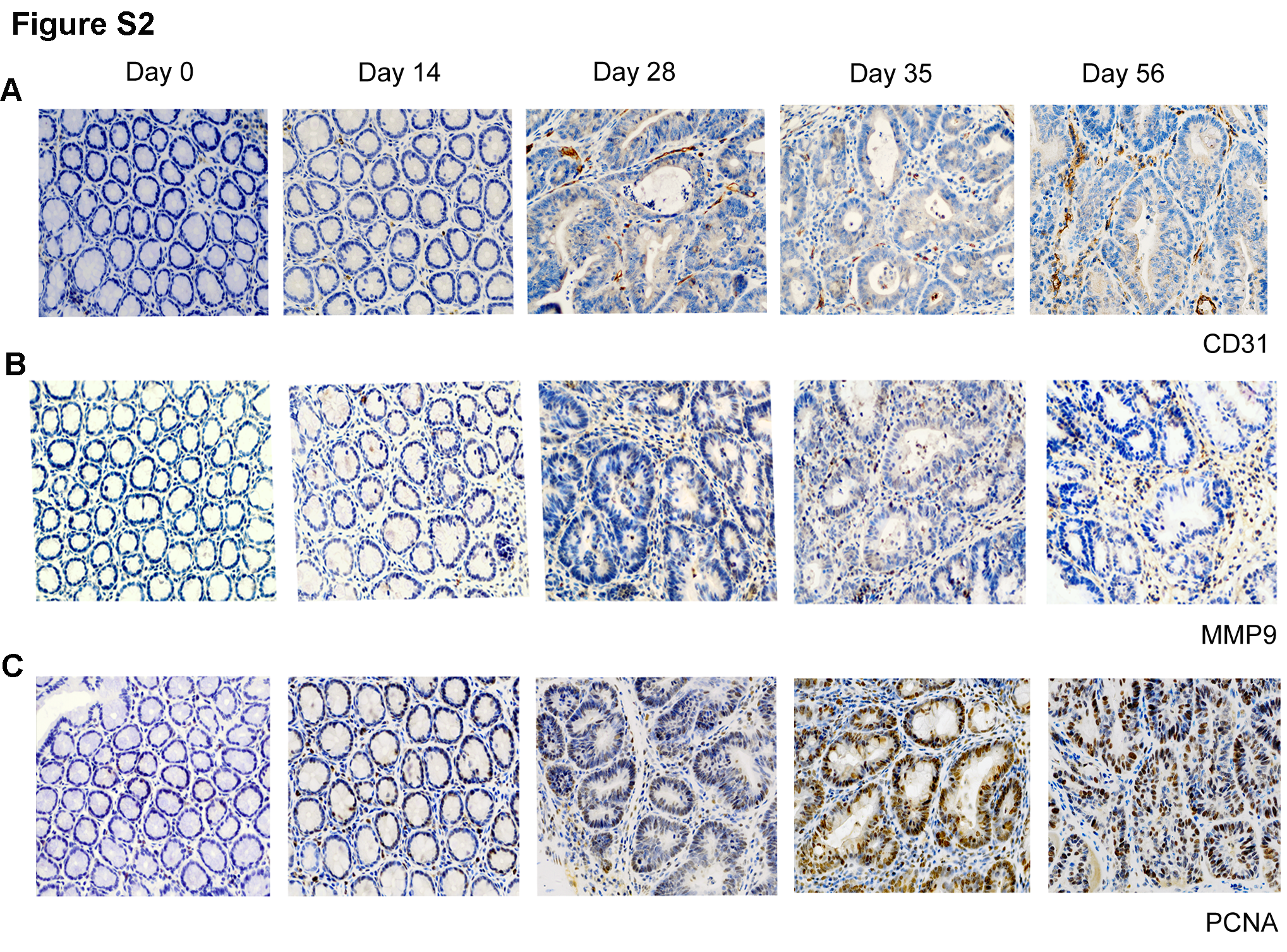

Supplement: Figure S2 — (A) Colon tissues were immunostained with anti-CD31 antibody. Representative results of 7 AOM and DSS-treated mice are shown. (B) Immunohistochemical analysis was performed using anti-MMP-9 antibodies as described in the Materials and Methods. Original magnification, 400×. (C) Representative results of immunohistochemical staining for PCNA in the colon tissues of 7 mice. Original magnification, 400×. (TIF) [file pone.0051848.s002.tif]

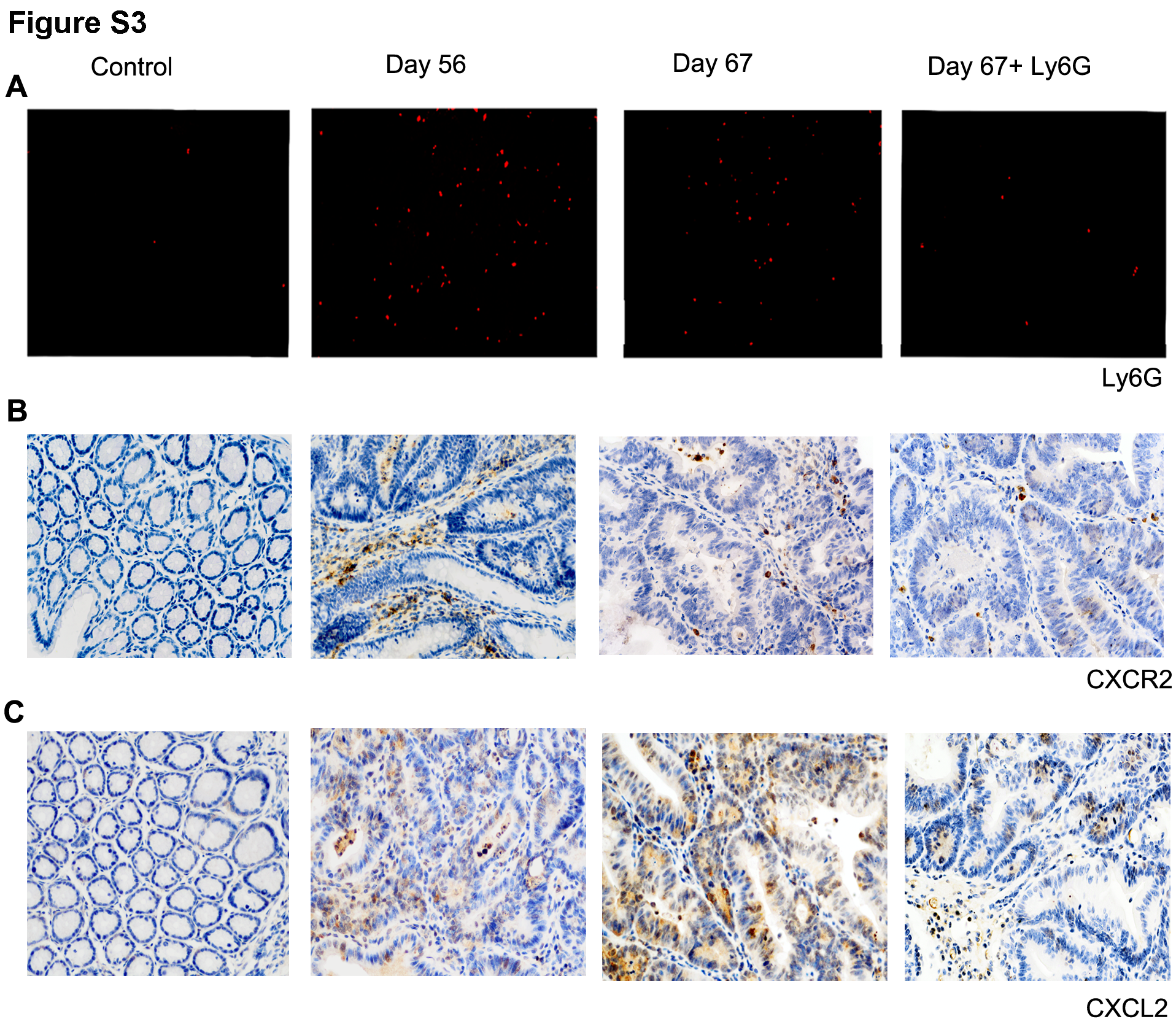

Supplement: Figure S3 — The effects of anti-Ly6G antibodies on neutrophil infiltration and CXCR2 and CXCL2 expression. Colon tissues were immunostained with anti-Ly6G (A), anti-CXCR2 (B), and anti-CXCL2 (C) antibodies as described in the Materials and Methods. Representative results of 5 mice are shown. (B and C) Original magnification, 400×. (TIF) [file pone.0051848.s003.tif]

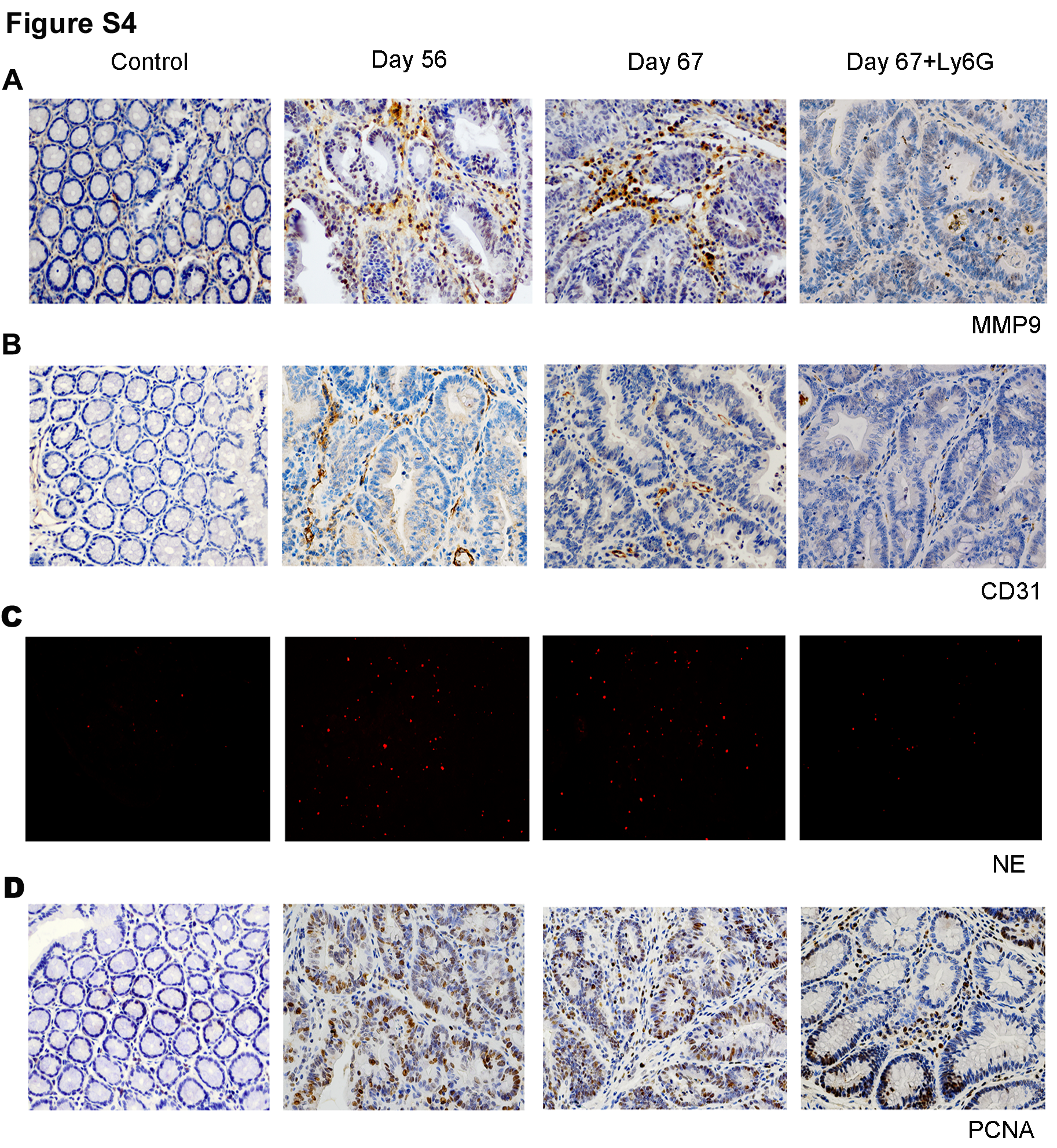

Supplement: Figure S4 — The effects of anti-Ly6G antibodies on neovascularization and cell proliferation. Cells were immunohistochemically stained with anti-MMP-9 (A), anti-CD31 (B), anti-PCNA (C), and anti-NE antibodies (D). Representative results of 5 mice are shown. Original magnification, 400×. (TIF) [file pone.0051848.s004.tif]
